# Comment on “Factors Associated with Recurrent Ulcers in Patients with Gastric Surgery after More Than 15 Years: A Cross-Sectional Single-Center Study”

**DOI:** 10.1155/2018/8356948

**Published:** 2018-12-16

**Authors:** Davide Giuseppe Ribaldone, Rinaldo Pellicano

**Affiliations:** ^1^Department of Medical Sciences, Division of Gastroenterology, University of Turin, C.so Bramante 88, 10126 Turin, Italy; ^2^Unit of Gastroenterology, Molinette Hospital, Corso Bramante 88, 10126 Turin, Italy

In a recent interesting study, Pantea et al. assessed the factors associated with recurrent gastric stamp ulcers more than 15 years after gastric surgery. The authors found that factors associated with ulcer recurrence were biliary reflux and alcohol consumption but not *Helicobacter pylori* (*H. pylori*) infection or treatment with drugs known to induce gastric lesions. To assess *H. pylori* status and for routine histology, at least three biopsy specimens were taken from the gastric remnant, anastomosis, and efferent side [1].

*H. pylori* is a slow-growing, microaerophilic, Gram-negative bacterium, usually acquired during childhood, whose natural habitat is the luminal surface of the gastric epithelium. *H. pylori* infection is accepted as the most important cause of gastritis and peptic ulcer in humans. Both *H. pylori* infection and aspirin/nonsteroidal anti-inflammatory drug (NSAID) use are the most relevant risk factors for the development of peptic ulcer and associated bleeding [2, 3]. *H. pylori* eradication prevents peptic ulcer recurrence and rebleeding [4]. Hence, detection and treatment of *H. pylori* remain the key-step in the strategy for long-term management of these patients. The methods used to diagnose *H. pylori* infection can be classified as invasive or noninvasive, the former being based on biopsy specimens obtained at endoscopy.

Pantea et al. [1] searched for *H. pylori* infection performing at least three biopsies. This could be a correct approach, but in patients currently treated with a proton pump inhibitor (PPI drug), false-negative results may occur [5]. Stopping PPIs two weeks before testing would allow the bacteria to repopulate the stomach and the tests previously negative could once again become positive [6]. Regarding the population included by Pantea et al., it is unclear if some of these patients took PPI drugs. This is crucial to better understand their negative data about the role of *H. pylori* infection. In any case, in patients with a gastric lesion and *H. pylori* negativity at histology, the evaluation of another method (for example, the stool test or the urea breath test) should have been considered, as a confirmatory test.

In conclusion, in the study by Pantea et al., a second test should have been done in case of *H. pylori* negativity to confirm this result.
